# Exploring mothers’ experiences of perinatal care in Cyprus: Babies Born Better survey

**DOI:** 10.1186/s12884-023-05800-5

**Published:** 2023-07-01

**Authors:** E. Hadjigeorgiou, M. Andreaki, I. Koliandri, A. Spyridou, M. C. Balaam, A. Christoforou

**Affiliations:** 1 Department of Nursing, School of Health Sciences, Cyprus University of Technology, Limassol, Cyprus; 2 School of Community Health and Midwifery, University of Central Lancashire, Preston, UK; 3 Department of Social & Behavioral Sciences and Health Science, European University Cyprus, Nicosia, Cyprus

**Keywords:** Experiences, Mothers, Cyprus, Intrapartum care, Childbirth, BBB

## Abstract

**Introduction:**

A positive perinatal experience facilitates a smooth transition to motherhood and enhances the development of a strong bond between mother and newborn, contributing to maternal and societal wellbeing. Given the medicalization of childbirth in Cyprus, the examination of mothers’ experiences of perinatal care becomes imperative.

**Aim:**

To investigate mothers’ experiences of care during the perinatal period and to identify factors related to the provision of maternal care that contribute to the interpretation of these experiences.

**Methods:**

The study draws on data from the European survey “Babies Born Better”, an online survey utilizing a mixed-methods approach to explore women’s experiences of maternity care across Europe. The study population were women who had given birth in Cyprus over a 5-year period (2013–2018). Quantitative data were analyzed using SPSS v22, while qualitative data were analyzed through inductive content analysis.

**Findings:**

A total of 360 mothers participated in the study. In rating their overall experience, 24.2% stated that they had a “*bad experience*”, 11.1% a “good experience”,13.9% a “*very good experience*”, and 13,3% a “*very bad experience*”. The top three sub-factors of the overall experience which received positive evaluation were “*Relationship with health care professionals*” (33.6%), “*Birth environment and care*” (11.4%), and “*Breastfeeding guidance*” (10.8%). The qualitative analysis yielded five themes: “Relationship with health care professionals”, “Breastfeeding establishment”, “Childbirth rights”, “Birth environment and services” and “Choice of mode of birth”.

**Conclusion:**

Mothers in Cyprus wish to have respectful maternity care. They need maternity health care professionals to respect their dignity and ask for evidence-based information with shared decision making. Mothers in Cyprus expect to have their childbirth rights safeguarded, to have better support from HCP, and to receive humanized care. The perinatal care provided in Cyprus needs significant improvements based on mothers’ needs and expectations.

## Introduction

The birth of a child is a pivotal point in the life of a woman and a family [22]. The experience of childbirth has been described as a complex, multidimensional, subjective phenomenon, characterized by a continuous alternation of negative and positive feelings with short and long-term physical and psychosocial effects for both the mother and the infant [1]. A positive childbirth experience can maintain or improve maternal wellbeing, empower mothers, and enhance mother–infant bonding [50, 51]. On the contrary, a negative childbirth experience increases the risk of postpartum psychological distress, postpartum depression, and post-traumatic stress symptoms [5].

A range of factors influence mothers’ experiences during childbirth [13, 29, 32]. Mukamurigo et al. [32] identified that sufficient information in relation to their care, respect, breastfeeding guidance (*p* < 0, 0006) and skin-to-skin contact had a positive effect on mothers’ experiences. Staff confidence, adequate analgesia and continuity of care also contributed positively. Deliktas et al. [13] identified the presence of relatives/friends during childbirth as having a positive impact on women’s experiences of childbirth. In Oosthuizen et al. [35] study, the key factors influencing mothers’ satisfaction with care received were the provision of reliable information, support of skin-to-skin contact, gentle vaginal examinations, permission to eat and drink during labor and adequate help and support for breastfeeding.

The mode of birth also affects mothers’ experiences of childbirth. Childbirth is a physiological process characterized by adaptation to a plethora of interconnected emotional, physical, and psychological changes, required for maternal adaptation and transition into the new role of motherhood. Mothers who had a normal birth (vaginal delivery) were more likely to feel a sense of maternal fulfillment, while mothers who gave birth by cesarean surgery were more likely to perceive the experience of childbirth as negative, especially in terms of their sense of control and bonding with the newborn. These mothers felt more concerned, expressed greater levels of insecurity, and lower self-confidence. Mothers who had a physiological birth expressed a greater sense of safety (74.7%) than mothers who gave birth by scheduled cesarean section (50%) with the lowest sense of safety being experienced by women undergoing an emergency cesarean (41.8%) [8]. Interventions during labor and birth affect women’s satisfaction levels and may prevent them from experiencing childbirth positively [50, 51]. Calik et al. 7 have found that among mothers who give birth with interventions, the level of satisfaction is relatively low: 139.59 ± 29.02 (≥ 150.5 = high satisfaction, < 150.5 = low satisfaction).

Perinatal care in Cyprus is obstetric-led and highly medicalized, characterized by high levels of interventions [20]. Indicatively, since 2014, Cyprus has been the country with the highest rates of caesarean surgery per capita in Europe. The perinatal healthcare system in Cyprus has been described as outdated and inefficient [22]. The investigation of women’s experiences and interpretations of care received during childbirth is crucial as it can provide objective and scientific insight. The country’s transformation into a multicultural society means that there are significant changes to the needs of mothers in relation to the past. The examination of positive childbirth experiences in the context under investigation and the factors associated with such experiences imparts added value that can be further utilized by healthcare professionals and policymakers to improve perinatal care. The introduction of the General Health System in Cyprus in 2019 and its ongoing evaluation provides ample opportunities for ensuring that all mothers in Cyprus receive quality perinatal care, regardless of nationality and cultural background. To the best of our knowledge, this is the first study that investigates the positive experiences of maternity care of women living in Cyprus.

## Methods

### The Babies Born Better survey

The present study is part of the Babies Born Better (BBB) online survey which was developed as part of the EU funded COST actions ISO907«Changing childbirth cultures and consequences» and IS1405 «Building Intrapartum Research Through Health – an interdisciplinary whole system approach to understanding and contextualizing physiological labor and birth». The BBB survey involves the participation of researchers from more than 38 countries in Europe and worldwide (https://www.babiesbornbetter.org/), with the aim to become a major resource for the improvement of maternal and childbirth care around the world. The data of what works, for whom and in what circumstances, will provide us insights into best practices for childbirth. The present article analyzes data collected in Cyprus during the second phase of the BBB study. Indicatively, this phase was conducted between March and August 2018 with the participation of more than 44 000 mothers worldwide [3]. The “Cyprus BBB” study, as it came to be called, collected data from mothers who gave birth in Cyprus within the 5-year period before recruitment into the study (2013–2018).

### The BBB questionnaire

The BBB survey questionnaire is composed of 17 questions designed to elicit sociodemographic characteristics (age, country and city of residence, country of origin, migration status and reason of immigration, duration of formal education, employment status, parity, marital status); most recent pregnancy information (duration of gestation, pregnancy-related problems, date of child’s birth, mode of birth); childbirth environment (place of birth, the type of health professionals in attendance); and, finally, perceptions and feelings of overall birth experience. The survey consisted of both close-ended and open-ended questions in which women were asked to provide their views about their experiences of care [6, 47].

The questionnaire was prepared by a group of researchers, and subsequently reviewed and improved by a wide range of stakeholders, including academics, activists, and people with diverse personal and professional backgrounds. The questionnaire was then translated into 23 languages for use across Europe and beyond. In Cyprus, the original questionnaire was used for English-speaking participants, whereas a Greek version for developed for Greek-speaking participants. The questionnaire was translated by native speakers (EH, AS and Greece team) into Greek and subsequently verified and refined using back translation to improve its reliability. Some transcultural adaptations were introduced in the items related to the Cyprus health system, birth setting, and birth professionals. The survey ran as an open online survey on SurveyMonkey® platform between March and August 2018.

### Data collection

The study utilized convenient sampling. The participants were recruited in two ways during the period of March-August 2018: (a) through social media, and (b) through the researchers’ own social networks. Invitations to participate in the study along with the survey link were extended through various social media channels, as well as personally through leaflets containing the survey link. The inclusion criteria were mothers aged 18 and above who had given birth in the previous 5 years and were residents of Cyprus, regardless of first language. The exclusion criteria omitted mothers who had not given birth in Cyprus and their age were below 18 years old.

Ethics approval for this international study was granted by the Ethics Committee of the University of Central Lancashire (UCLAN) in the UK (Ethics Committee BuSH 222 & STEMH 449). All data was stored and handled in accordance with the General Data Protection Regulation, the UK Data Protection Act (1998), as well as the University of Central Lancashire (UK) guidelines. The front page of the survey provided potential participants with information about confidentiality, consent, data protection and the scope and aims of the survey. Based on this information, participants consented to participate by choosing to progress beyond this front page.

### Data analysis

The analysis of the data was carried out using descriptive and correlation statistics methods with the help of the Statistical Package of Social Sciences SPSS 22 (Statistical Package of Social Sciences IBM-SPSS 22). The level of statistical significance was set in all cases at 5%. Chisquare (χ^2^) correlation was explored between “Feelings about childbirth experience” with “Type of childbirth”. Qualitative data were analyzed through inductive content analysis, adhering to the following steps: (1) reading the full set of data to obtain an overview; (2) codification of each answer by combining inductive procedures; (3) identification of themes and categorization (4) intra code and intercode comparison and subsequent recodification to ensure the internal consistency of codes and subcategories; (5) merging of subcategories to themes. The same process was followed independently by two researchers and any discrepancies regarding themes were discussed until consensus was reached. This is a systematic and objective method designed to describe in depth a phenomenon in order to make it more understandable [10]. It is also an appropriate approach for analyzing answers to open-ended questions and was therefore determined as a suitable method for this study [15].

## Results

### Sociodemographic characteristics

A total of 360 mothers fulfilling the aforementioned inclusion criteria completed the BBB questionnaire. Indicatively, the corresponding population was estimated at 9223 births in 2017, requiring a sample of 369 participants (Cl 95%, 5%). The detailed demographic characteristics of the participants are presented in Table 1. Of the total number of participants, 72% gave birth in a private clinic, in comparison to 28% who gave birth in a public hospital. It is typical for Cyprus that more mothers prefer to give birth in private settings [20]. In terms of mode of birth, the proportion of mothers who gave birth vaginally and by cesarean surgery is identical, at 39.2% for each group. This means the 39.2% rate of cesarean surgery of mothers participating in the survey, is lower than the officially published data (53.6% report of [31]).

**Table 1:** Sociodemographic characteristics

	*N* = 360	%
**Age**		
22–27 years	23	6,4%
28–33 years	155	43,1%
34–39 years	152	42,2%
>40 years	27	7,5%
**Place of Residence**		
Nicosia	201	55,8%
Limassol	53	14,7%
Larnaca	49	13,6%
Paphos	13	3,6%
Other	20	5,6%
**Nationality**		
Cypriot	295	81,9%
Non-Cypriot	65	18,1%
**Marital status**		
Married/ In a relationship/ Cohabiting	345	95,8%
In a relationship/Not cohabiting	2	0,6%
Single	8	2,2%
Divorced	2	0,6%
**Educational**		
Post-secondary	12	3,3%
High School/College	94	26,1%
University	244	67,7%
**Employment**		
Employed	307	85,3%
Unemployed	35	9,7%
Self-employed	5	1,4%
University student	3	0,8%

### Mother’s experiences of perinatal care

#### Ranking of positive experiences

In the question asking participants to select from a list of options and rank “the three positive experiences of your care (with the first being the most important for you)” it emerged that the most frequently chosen answer, as well as the one most often rated at utmost importance for the women, was “the relationship with health professionals” (33.6%). “Breastfeeding guidance” ranked second in importance, selected at a rate of 10.8%, and of third importance, was the “Birth environment and care”, with 11.4% of participants choosing this as one of the three factors. The least frequently selected positive experience was mode of birth, chosen by only 1.7% of respondents. (see Table 2).

**Table 2:** Ranking of positive experiences

	**First Positive Experience**	**Second Positive Experience**	**Third Positive Experience**
**Relationship with Healthcare professionals**	33.6% (121)	24.7% (89)	9.7% (35)
**Breastfeeding guidance**	8.9% (32)	10.8% (39)	5.6% (20)
**Childbirth rights**	6.4% (23)	3.1% (11)	2.2% (8)
**Birth Environment and care**	9.7% (35)	8.3% (30)	11.4% (41)
**Mode of Birth**	4.4% (16)	1.9% (7)	1.7% (6)
**None**	5.6% (20)	0.8% (3)	0.8% (3)

#### Recommendations for improvement

In the questionnaire section on recommendations for improvement of care provided, the most frequently selected answers by mothers were “choice of type of childbirth” (10.6%), “Relationship with Health care professionals” (8.1%), and “Birth environment and care” (6.4%), followed by “Breastfeeding guidance” and “Childbirth rights” The majority of the sample had no specific recommendations to suggest (see Table 3).

**Table 3:** Recommendations for improvement

Recommendations	*Ν* = 360	%
**Relationship with Health care professionals**	**29**	**8,1%**
Breastfeeding guidance	18	5%
Childbirth rights	16	4,4%
**Birth Environment and care**	**23**	**6,4%**
**Mode of Birth**	**38**	**10,6%**
None	75	20,8%

### Mode of birth

#### Type of childbirth and feelings about childbirth experience

“Feelings about childbirth experience” correlated significantly with “Mode of birth”, the respondents, 21.7% - of women who experienced a normal birth stated that they had a “very good experience”. By comparison, of mothers who gave birth with a planned cesarean section, 13.6% - said they had a “very good experience”. The mothers who gave birth by emergency cesarean section 10.3% chose the answer “very good experience” to the corresponding question, while of those who gave birth with ventouse intervention 35% had a very good experience in correlation to the women who had an emergency cesarean section (*p*-value = 0.03, chi-square 22.342, df: 12).

The option “quite a bad experience”, was chosen by 16.9% mothers who gave birth normally, 21.2% of mothers who gave birth by scheduled cesarean surgery, 17.2% of mothers who gave birth by emergency cesarean and 15% of the mothers who gave birth with ventouse intervention. The results indicate that mothers who gave birth normally or with ventouse assistance, had a better experience compared to mothers who gave birth through planned or emergency cesarean surgery, at the level of statistical significance (*p*-value = 0.03, chi-square 22.342, df: 12).

The birth experience was also found to be correlated with (1) the type of birth, where it was shown that mothers who gave birth by vaginal birth more often reported having a “very good experience” (21.7%) than those who gave birth by cesarean section (13.6%) (*p* = 0.03) and (2) interventions in childbirth, where “very good birth experience” had women with “no intervention” by 26.2%, while 8.3% with “accelerated labor” (*p* = 0.001).

### Relationship with health care professionals

#### Advice to a loved one

In one section, mothers were asked to advise a loved one about giving birth in the same clinic, giving reasons as to why or why they did not recommend the particular venue. Only 19.4% of participants answered this question. Of those who responded, 61.7% said they would recommend giving birth in the same location, with the most frequently cited reason being the good “relationship with health care professionals” (39,2%). Some mothers (6.4%) did not recommend birthing in the same venue, also citing the same reason (Table 3).

#### Birth environment and care

While the 23,1% (83/360) answered on the question about the birth environment where the 6,9% reported positive comments, the 10,8% rated their experience of birth environment and care as negative. Lastly, 5,3% reported neutral comments.

### Qualitative data

#### Maternal experiences during childbirth

From the inductive analysis of the data on the open-ended questions five broad themes emerged and each theme was divided into sub-themes (Table 4).

**Table 4:** Thematic areas emerging from the study

**Thematic Areas**	**Sub-themes**
**Relationship with health professionals**	The behavior of health professionals towards mothers
	Midwifery model vs medical model of care
	Experience and expertise/ professionalism of health professionals
**Establishment of breastfeeding**	Education / Promotion of breastfeeding
	Rooming in
	Imitate of Breastfeeding and skin to skin
**Childbirth Rights**	Respect of maternal choices and desires
	Consent after being informed
**Birth environment**	Equipment
	Single en-suite rooms
	Friendly and Beautiful Environment
	Cleanliness
	Good food
	Easy access to NICU
**Choice of Birth mode**	Promotion of vaginal birth
	Avoidance of interventions

The thematic areas were: 1. “Relationship with health professionals”, 2. “Establishment of breastfeeding”, 3. “Childbirth rights”, 4. “Birth environment and care”, 5. “Choice of birth mode”

#### Relationship with healthcare professionals (HCPs)

The relationship between mothers and HCP was a key factor, contributing to the overall quality of the childbirth experience. Mothers stressed the importance of the HCP experience, and significantly valued the expertise and the support provided during labour and birth. Patience, courtesy, and politeness shown by HCPs during labour and birth were deeply appreciated.

*Experience and expertise/ professionalism of HCP.*

The experience and expertise of the HCPs was also identified as an important for building a relationship of trust, that made mothers feel safe:

“*My health care professionals were experienced; they have the necessary knowledge…I feel safe as I trust my doctor and my midwife during pregnancy and especially during childbirth.*” (*ID 6841158169)*

When mothers were asked about potential changes that would improve their care, interesting examples of very poor care emerged:

*“Doctors are thinking of money. There is no cooperation between mothers, doctors and nurses and it seems that they do not care much” (ID6890272490)*

Another mother said that she would not advise any woman to give birth in the same setting she had given birth because of the unprofessional behavior of some HCPs:

*“They didn’t care for me at all and I felt like I was a money machine for them” (ID 6733027836).*

Mothers also commented on the different models of care and on the levels of support provided by midwives and obstetricians:

*“My second birth took place only in the presence of midwives and my husband. Everything was very natural and better than my first baby where the doctor was present…the doctor was anxious, in a hurry and did not provide the same guidance as midwives” (ID 6732499265).*

One mother made a special reference to the midwives’ behavior:

“*Midwives were gentle and helpful during labor and that made me feel comfortable*” (*ID 6733569197)*

Interestingly, several mothers commented that they were satisfied with the continuity of care offered by midwives during labor and birth, in contrast to the care offered by the obstetrician:

*“I appreciate the overnight support from my midwife, in contrast to the brief presence of the doctor at birth” (ID 6781575650).*

#### Establishment of breastfeeding

Many mothers mentioned that initiating and sustaining breastfeeding was of critical importance and an integral component of their childbirth experience. Skin-to-skin care and the first hours after birth were perceived by mothers as the most important steps for the establishment of breastfeeding. An environment that promotes both breastfeeding and the mother’s involvement in newborn care was a significant factor in overall satisfaction with the childbirth experience:

“*Midwives were continuously promoting breastfeeding and were giving advice”(ID 6732232003).*

On the other hand, some mothers mentioned that there were HCPs who did not support breastfeeding, offered conflicting advice, and interrupted mothers during breastfeeding. Attention to breastfeeding was elaborated on by some mothers who referred to the “10 Steps to Successful Breastfeeding Initiation” [50, 51]. These women described their failed efforts at “rooming-in” and “skin-to-skin, right after birth” because of policies at their birth venue:

*“I asked them to help me with skin-to-skin after birth, but they did not listen… then I asked them for rooming-in but they said to me that for the baby’s best interest the baby must stay in the nursery” (ID 6785652175).*

### Childbirth rights

Involvement or lack of involvement in decisions surrounding birth mode and care was one of the main issues highlighted by participants through the survey, and, as such, may have a significant bearing on mothers’ overall experience. Mothers emphasized that an important element of their care was that their choices and desires were heard and respected. One mother described how difficult it was for her to give birth in the way she wanted to:

“*My choice to give birth with VBAC was a battle for me with my doctor because VBAC is not allowed at this maternity clinic.” (ID 6864013384)*

Another mother described her choices as being only partially met, noting that:

*“Most of the time they did not allow me to make a simple decision” (ID 6841647617).*

Mothers describe with positive remarks, the respectful care they received:

*“The doctor respected me and always asked for my consent, he was very supportive”. (ID 6785652157)*

A mother commented that the midwives were very supported, but that this was a pleasant surprise, suggesting that this was not an expected situation:

*“Midwives explained more than I would expect”.* *(ID6747664260).*

It was common for mothers to express satisfaction at being able to choose to have their partner present during birth:

*“That was a pleasure to have my husband by my side, during labor” (ID 6793222090)*

Some mothers mentioned that their childbirth rights were not consistently safeguarded. The lack of respect and non-response towards their expressed wishes regarding birth mode, privacy, and choice of persons present in the labour room, as well as poor communication between mothers and HCPs were among the examples given. One mother characteristically mentioned:

“When *the doctor first examined me at birth…she shouted at me that I must follow her orders. I was very shocked, no one ever spoke to me like that… I could not speak between the contractions, and I was alone, and I could not defend myself. I was very hurt and felt myself in a vulnerable position*” *(ID 6785652175)*

#### Birth environment and care

Mothers identified the birth environment as important, commented positively on modern and clean surroundings, single and cozy rooms, access to a private toilet, and close proximity to the NICU. Participants noted:

*“There was exemplary hygiene.” (ID 6924710149).*

*“There were balls that could help me with birth” (ID 6737100934).*

*“The environment in the clinic was very harmonious and modern (with low lighting, music)” (ID 6733392130).*

*“That it was close to the NICU.” (ID 6732173142).*

However, for some mothers the birth environment was a negative aspect of their experience with some commenting on old equipment, lack of private rooms, inadequate room cleanness, and lack of options for labour pain relief. Some women also commented on the lack of choice in birth venues and the unavailability of home birth in Cyprus, while others commented on the lack of provision of prenatal classes or the high cost of childbirth.

*“The equipment was very old” (ID 6746895859).*

*“They could have been less machinery in the room and more privacy” (ID 6781571507)*

*“There should have been better food (which is important after so many hours of birth and considering the cost is very high) and better care to the mother” (ID 6787210803).*

#### Choice of birth mode

Choice of birth mode was described by mothers as an important element of their care and as one which enhanced their experience. Many women emphasized their wish to avoid unnecessary interventions, including induction and cesarean section in the absence of serious complications. Some women expressed feeling proud of themselves for having given birth naturally, while many mothers mentioned that they chose their birth venue based on the venue’s reputation in promoting and prioritizing normal birth. Some women also reflected on the high medicalization of childbirth in Cyprus, making the experience of childbirth less pleasant:

*“If it is possible for a mother to leave Cyprus and give birth anywhere else in the world, then book a trip immediately. It is unbelievable the degree of medicalization of care here” (ID 6787210803)*

*“In case that you want a natural birth without interventions, then you should avoid the hospitals and maternity clinics in Cyprus, including the one where I gave birth” (ID 6781575652)*

## Discussion

This study provides evidence on how mothers giving birth in the Republic of Cyprus perceive their experiences. Support by HCPs, respect of the mothers’ wishes, and active involvement in childbirth were associated with a positive experience. The expertise and professionalism of HCPs also contributed to a positive experience along with continuity-of-care and implementation of good maternity practices within a friendly, comfortable, and clean environment.

The development and nurturing of relationships of trust between HCPs and pregnant women have emerged as highly significant. Positive emotions increased during childbirth if mothers were respected and treated with kindness. These results are congruent with Raboteg-šarić et al.’s 38 study, which indicated that HCPs general behavior was perceived by mothers as one of the most important attributes for experiencing childbirth positively. Provision of quality care and professionalism were also the main factors that emerged in a study on the contribution of HCPs to the formation of positive childbirth experiences in the Austrian B3 study [29].

An interesting result was the extent to which HCPs’ behavior and the medicalised nature of maternity care in Cyprus created a negative perception of childbirth experience. In particular, the apathetic behavior of some HCPs, as well as the lack of respect, courtesy, and knowledge, did not support the development of a pleasant experience. Insufficient information, failure to elicit consent to any intervention (eg., giving the infant formula milk) and lack of respect for women’s values and wishes, including the right to choose how to give birth, were associated with mothers’ dissatisfaction with care, which resonates with the findings of the study by Shabila et al. [43]. The behavior of HCPs needs to be improved, unnecessary interventions must be avoided, and birth must be humanized [45].

The factor least often chosen by participants as contributing to a positive birth experience was birth mode. It is interesting to ask whether this means that overall, women were least happy with their mode of birth. This is a topic which warrants more in-depth examination, particularly given the high levels of medicalised birth intervention in Cyprus. The failure to facilitate and promote normal/physiological childbirth, as well as the high rates of unnecessary cesarean surgery and other interventions, have been found to contribute to negative perceived experiences of care, as evidenced in the research of Pazandeh et al. [36] on the increased medicalisation of childbirth. The healthcare system in Cyprus does not pay enough attention to the needs for laboring mothers’ feelings of safety and support. Mothers often describe a lack of a humane approach, lack of support, bonding and safety, and insufficient quality presence of the doctors. Abuse of power and psychological manipulation are also evident, with women feeling that they were mere sources of income for doctors, being exploited while in a vulnerable condition.

Many mothers ask for normal childbirth, for the avoidance of unnecessary interventions, and for the performance of cesarean surgery only when absolutely necessary. The relationship between mothers, HCPs and the birth environment needs improvement. Mothers wish to see improvements in the birth environment, and in particular the development of environments which are friendly, provide options for pain relief, including natural methods such as music, and support the presence of a partner during labor and birth. In addition, access to single rooms with an ensuite toilet and bathtub in a clean and an aesthetically pleasing maternity ward is recommended. The ability to opt for other means of childbirth such as water birth, was also expressed as desirable. The recommendations for improvement reported by Croatian mothers in Raboteg-šarić et al.’s 38 study were similar,the development of an environment facilitating natural birth without interventions, where mothers could have more control over their experience and more support and understanding from HCPs was emphasized. Austrian mothers who participated in the BBB survey similarly suggested improving the birth environment and care (23%), a more positive experience (22%) and providing more personalized care (21%) [29].

Correlations between type of childbirth and feelings about childbirth experience was found. Women that had natural childbirth had reported a positive childbirth experience in relation to the women that had a scheduled or emergency cesarean section. Similar findings were reported from Carquillat et al. [8], where they explored how the type of childbirth was associated with the childbirth experience. It was found that women that had cesarean section were more likely to experience negative feelings, like worrying and insecurities. The women also reported that the first moments with the newborns were not as fulfilling in contrast to the women that had a natural childbirth. The negative experience of childbirth is related to the medicalization of childbirth with increased intervention. In a Canadian study, Chalmers and Dzakpasu 9 found that childbirth interventions are an aggravating factor in shaping a positive experience, with a statistically significant correlation with positive experience for mothers who gave birth normally. Further to the above, women in Cyprus need better support for breastfeeding establishment, implementation of skin-to-skin in all maternity hospitals and promotion of rooming-in.

Similar results were found in the other countries BBB survey was carried out; specifically the Norwegian team reported that women are in need of a more compassionate and respectful maternity care with continuity of care where the socio-cultural and psychological impacts of care are utmost importance for the women during childbirth [47]. In addition, the BBB survey in Ireland reported the need for women to have an adequate relationship with their healthcare professionals that respect and support not only their decisions but supporting shared decision making [34]. These findings are also supported by the results of the study in Spain where the main findings report the women’s need for shared decision-making with their healthcare professional, also the need for a change in natural childbirth facilitation without interventions and unnecessary practices was highlighted [6].

### Strengths and limitations

The mothers that participated in the BBB survey study were 39.2% that gave birth by cesarean surgery; while the official published data for Cyprus reported that 53.6% of women had cesarean therefore, it remains unknown whether women that had cesareans were generally under-represented (Report of Ministry of Health [31]). Nonetheless, the perceived care of mothers allowed us to gain insights to their perspectives considering the birth setting, mode of birth and experiences of birth which is crucial in order to establish a more woman-centered care. While similar studies have been conducted in various countries around the world, either with a quantitative or qualitative design [8, 40], the major strength of the “Babies Born Better” project is that it can lead to a constructive comparison of perinatal care among European countries. Practices that have been shown to yield positive results in other European countries can easily adapted, piloted, and implemented in the Cypriot context [20].

## Conclusion

The most significant finding from this study is the need for changes in perinatal care in Cyprus. The improvement of the maternal healthcare system and the promotion and facilitation of positive experiences of labor and childbirth are imperative. Women in Cyprus expect to have their rights in childbirth safeguarded, and to receive respectful care and better support from HCPs, as well as evidence-based care during labor, birth, and breast-feeding establishment. The experiences of mothers should inform public and political debates on the quality of maternity care in Cyprus and the findings of the present study provide valuable insight into this topic.

## Data Availability

The data that support the findings of this study are available from the corresponding author upon reasonable request.

## Abbreviations

BBB: Babies Born Better
WHO: World Health Organization
HCP: Healthcare professional
